# [(*Z*)-*O*-Ethyl *N*-(4-chloro­phen­yl)thio­carbamato-κ*S*](triphenyl­phosphine-κ*P*)gold(I) dichloro­methane hemisolvate

**DOI:** 10.1107/S1600536810018179

**Published:** 2010-05-22

**Authors:** Primjira P. Tadbuppa, Edward R. T. Tiekink

**Affiliations:** a Department of Chemistry, National University of Singapore, Singapore 117543; b Department of Chemistry, University of Malaya, 50603 Kuala Lumpur, Malaysia

## Abstract

The Au^I^ atom in the title compound, [Au(C_9_H_9_ClNOS)(C_18_H_15_P)]·0.5CH_2_Cl_2_, exists within a slightly distorted linear geometry defined by an *S*,*P* donor set [S—Au—P angle = 178.01 (4)°]; a close intra­molecular Au⋯O contact [2.964 (4) Å] also occurs. In the crystal structure, mol­ecules are linked into supra­molecular chains propagating along [010] by C—H⋯N, C—H⋯S and C—H⋯π inter­actions. The solvent mol­ecule is disordered about a twofold rotation axis.

## Related literature

For the structural systematics and luminescence properties of phosphinegold(I) carbonimidothio­ates, see: Ho *et al.* (2006 ▶); Ho & Tiekink (2007 ▶); Kuan *et al.* (2008 ▶). For the synthesis, see: Hall *et al.* (1993 ▶). 
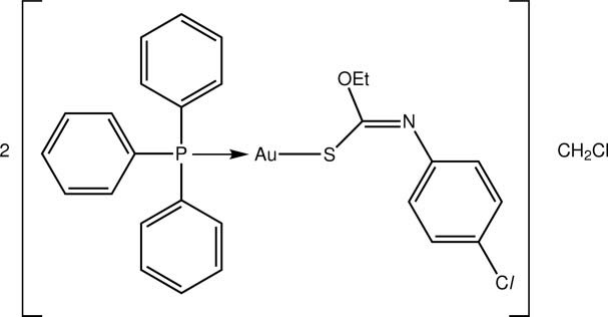


## Experimental

### 

#### Crystal data

[Au(C_9_H_9_ClNOS)(C_18_H_15_P)]·0.5CH_2_Cl_2_
*M* *_r_* = 716.40
Monoclinic, 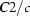

*a* = 30.5163 (16) Å
*b* = 8.5881 (5) Å
*c* = 21.0518 (12) Å
β = 101.054 (1)°
*V* = 5414.8 (5) Å^3^
*Z* = 8
Mo *K*α radiation
μ = 5.79 mm^−1^
*T* = 223 K
0.15 × 0.15 × 0.13 mm

#### Data collection

Bruker SMART CCD diffractometer
Absorption correction: multi-scan (*SADABS*; Bruker, 2000 ▶) *T*_min_ = 0.672, *T*_max_ = 1
18509 measured reflections
6214 independent reflections
5381 reflections with *I* > 2σ(*I*)
*R*_int_ = 0.031

#### Refinement

*R*[*F* ^2^ > 2σ(*F* ^2^)] = 0.032
*wR*(*F* ^2^) = 0.092
*S* = 1.10
6214 reflections
306 parameters
H-atom parameters constrained
Δρ_max_ = 1.73 e Å^−3^
Δρ_min_ = −1.87 e Å^−3^

### 

Data collection: *SMART* (Bruker, 2000 ▶); cell refinement: *SAINT* (Bruker, 2000 ▶); data reduction: *SAINT*; program(s) used to solve structure: *PATTY* in *DIRDIF92* (Beurskens *et al.*, 1992 ▶); program(s) used to refine structure: *SHELXL97* (Sheldrick, 2008 ▶); molecular graphics: *ORTEP-3* (Farrugia, 1997 ▶) and *DIAMOND* (Brandenburg, 2006 ▶); software used to prepare material for publication: *publCIF* (Westrip, 2010 ▶).

## Supplementary Material

Crystal structure: contains datablocks global, I. DOI: 10.1107/S1600536810018179/hb5453sup1.cif

Structure factors: contains datablocks I. DOI: 10.1107/S1600536810018179/hb5453Isup2.hkl

Additional supplementary materials:  crystallographic information; 3D view; checkCIF report

## Figures and Tables

**Table 1:** Selected bond lengths (Å)

Au—P1	2.2578 (11)
Au—S1	2.3064 (11)

**Table 2:** Hydrogen-bond geometry (Å, °) *Cg*1 is the centroid of the C22–C27 ring.

*D*—H⋯*A*	*D*—H	H⋯*A*	*D*⋯*A*	*D*—H⋯*A*
C21—H21⋯N1^i^	0.94	2.55	3.310 (6)	138
C26—H26⋯S1^ii^	0.94	2.86	3.738 (6)	156
C7—H7⋯*Cg*1^i^	0.94	2.96	3.784 (5)	147

Symmetry codes: (i) 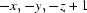; (ii) 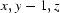.

**Fig. 1.**
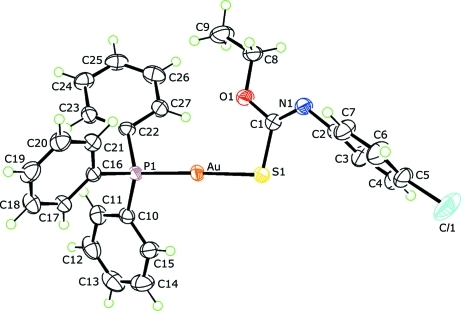
Molecular structure of (I) showing displacement ellipsoids at the 50% probability level. The solvent CH2Cl2 molecule of crystallisation is omitted.

**Fig. 2.**
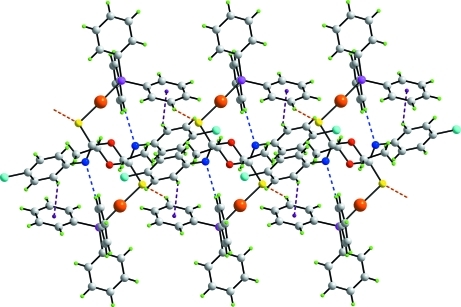
A view of the supramolecular chain in (I), aligned along the b axis, mediated by C–H···N (blue), C–H···S (orange), and C–H···π contacts (purple) shown as dashed lines.

**Fig. 3.**
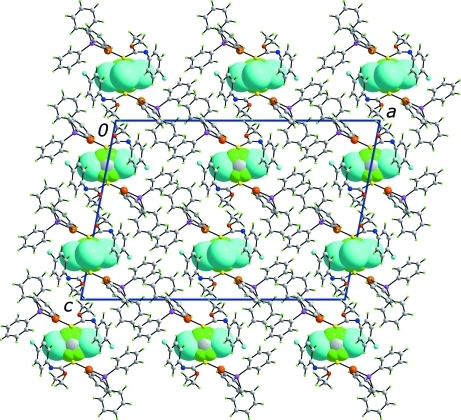
A view in projection down the b axis of the crystal packing in (I), highlighting the channels in which reside the (disordered) CH2Cl2 molecules (shown in space filling mode).

## supplementary crystallographic information

### Comment

The structure of the title compound, (I), was investigated in the context of a study of molecules with the general formula R_3_PAu[SC(OR')═NR''], for R, R' and R'' = alkyl and aryl, of interest in terms of crystal engineering endeavours (Ho *et al.*, 2006; Ho & Tiekink, 2007; Kuan *et al.*, 2008).

The nearly linear *SP* coordination geometry observed for the Au atom in (I), Fig. 1, is defined by phosphine and thiolate ligands, Table 1. The small deviation from the ideal linearity [S—Au—P = 178.01 (4) °] is related to a short intramolecular Au···O contact [2.964 (4) Å].

The major feature of the crystal packing is the presence of C–H···N (leading to centrosymmetric dimers), C–H···S and C–H···π interactions that lead to the formation of supramolecular chains along the *b* axis, Fig. 2 and Table 2. Chains are arranged to form channels in which reside the (disordered) CH_2_Cl_2_ molecules, Fig. 3.

### Experimental

Compound (I) was prepared following the standard literature procedure from the reaction of Ph_3_AuCl and EtOC(═S)N(H)(C_6_H_4_Cl-4) in the presence of NaOH (Hall *et al.*, 1993).  Yellow blocks of (I) were obtained by the slow evaporation of a CH_2_Cl_2_/hexane (3/1) solution held at room temperature.

### Refinement

The H atoms were geometrically placed (C—H = 0.94–0.98 Å) and refined as riding with *U*_iso_(H) = 1.2-1.5*U*_eq_(C). The maximum and minimum residual electron density peaks of 1.73 and 1.87 e Å^-3^, respectively, were located 0.68 Å and 0.52 Å from the Cl2 atom. The solvent CH_2_Cl_2_ molecule (modelled isotropically) was disordered about a 2-fold axis of symmetry with the C and one Cl atom lying on the axis.

### Crystal data

**Table:**

[Au(C_9_H_9_ClNOS)(C_18_H_15_P)]·0.5CH_2_Cl_2_	*F*(000) = 2792
*M**_r_* = 716.40	*D*_x_ = 1.758 Mg m^−^^3^
Monoclinic, *C*2/*c*	Mo *K*α radiation, λ = 0.71069 Å
Hall symbol: -C 2yc	Cell parameters from 6681 reflections
*a* = 30.5163 (16) Å	θ = 2.5–29.1°
*b* = 8.5881 (5) Å	µ = 5.79 mm^−^^1^
*c* = 21.0518 (12) Å	*T* = 223 K
β = 101.054 (1)°	Block, yellow
*V* = 5414.8 (5) Å^3^	0.15 × 0.15 × 0.13 mm
*Z* = 8	

### Data collection

**Table:**

Bruker SMART CCD diffractometer	6214 independent reflections
Radiation source: fine-focus sealed tube	5381 reflections with *I* > 2σ(*I*)
graphite	*R*_int_ = 0.031
ω scans	θ_max_ = 27.5°, θ_min_ = 1.4°
Absorption correction: multi-scan (*SADABS*; Bruker, 2000)	*h* = −39→38
*T*_min_ = 0.672, *T*_max_ = 1	*k* = −6→11
18509 measured reflections	*l* = −27→27

### Refinement

**Table:**

Refinement on *F*^2^	Primary atom site location: structure-invariant direct methods
Least-squares matrix: full	Secondary atom site location: difference Fourier map
*R*[*F*^2^ > 2σ(*F*^2^)] = 0.032	Hydrogen site location: inferred from neighbouring sites
*wR*(*F*^2^) = 0.092	H-atom parameters constrained
*S* = 1.10	*w* = 1/[σ^2^(*F*_o_^2^) + (0.05*P*)^2^ + 9.3001*P*] where *P* = (*F*_o_^2^ + 2*F*_c_^2^)/3
6214 reflections	(Δ/σ)_max_ = 0.001
306 parameters	Δρ_max_ = 1.73 e Å^−^^3^
0 restraints	Δρ_min_ = −1.87 e Å^−^^3^

### Special details

**Table:**

Geometry. All s.u.'s (except the s.u. in the dihedral angle between two l.s. planes) are estimated using the full covariance matrix. The cell s.u.'s are taken into account individually in the estimation of s.u.'s in distances, angles and torsion angles; correlations between s.u.'s in cell parameters are only used when they are defined by crystal symmetry. An approximate (isotropic) treatment of cell s.u.'s is used for estimating s.u.'s involving l.s. planes.
Refinement. Refinement of *F*^2^ against ALL reflections. The weighted *R*-factor wR and goodness of fit *S* are based on *F*^2^, conventional *R*-factors *R* are based on *F*, with *F* set to zero for negative *F*^2^. The threshold expression of *F*^2^ > 2σ(*F*^2^) is used only for calculating *R*-factors(gt) etc. and is not relevant to the choice of reflections for refinement. *R*-factors based on *F*^2^ are statistically about twice as large as those based on *F*, and *R*- factors based on ALL data will be even larger.

### Fractional atomic coordinates and isotropic or equivalent isotropic displacement parameters (Å^2^)

**Table:**

	*x*	*y*	*z*	*U*_iso_*/*U*_eq_	Occ. (<1)
Au	0.082143 (5)	0.03531 (2)	0.391240 (8)	0.02794 (7)	
Cl1	−0.15255 (7)	0.6401 (2)	0.23755 (9)	0.0849 (7)	
S1	0.01688 (4)	0.16692 (14)	0.35074 (6)	0.0351 (3)	
P1	0.14730 (3)	−0.08554 (13)	0.43121 (5)	0.0251 (2)	
O1	−0.00607 (11)	−0.0698 (4)	0.41402 (17)	0.0367 (8)	
N1	−0.06288 (12)	0.1010 (5)	0.38198 (19)	0.0344 (9)	
C1	−0.02287 (15)	0.0627 (5)	0.3836 (2)	0.0301 (9)	
C2	−0.08159 (14)	0.2323 (6)	0.3475 (2)	0.0317 (9)	
C3	−0.08667 (17)	0.2412 (7)	0.2804 (2)	0.0416 (11)	
H3	−0.0754	0.1612	0.2577	0.050*	
C4	−0.10818 (18)	0.3672 (7)	0.2469 (2)	0.0460 (13)	
H4	−0.1114	0.3727	0.2017	0.055*	
C5	−0.1248 (2)	0.4838 (7)	0.2801 (3)	0.0486 (14)	
C6	−0.1206 (2)	0.4782 (7)	0.3461 (3)	0.0513 (15)	
H6	−0.1321	0.5586	0.3684	0.062*	
C7	−0.09900 (18)	0.3514 (7)	0.3793 (2)	0.0436 (12)	
H7	−0.0962	0.3465	0.4245	0.052*	
C8	−0.03693 (17)	−0.1648 (6)	0.4414 (3)	0.0438 (12)	
H8A	−0.0639	−0.1860	0.4090	0.053*	
H8B	−0.0456	−0.1117	0.4783	0.053*	
C9	−0.0126 (2)	−0.3152 (7)	0.4631 (3)	0.0581 (16)	
H9A	−0.0320	−0.3831	0.4821	0.087*	
H9B	0.0141	−0.2922	0.4951	0.087*	
H9C	−0.0042	−0.3663	0.4262	0.087*	
C10	0.18807 (15)	−0.0764 (6)	0.3788 (2)	0.0291 (9)	
C11	0.22367 (16)	−0.1789 (7)	0.3848 (2)	0.0387 (11)	
H11	0.2262	−0.2602	0.4152	0.046*	
C12	0.25552 (17)	−0.1628 (8)	0.3465 (3)	0.0494 (14)	
H12	0.2799	−0.2317	0.3512	0.059*	
C13	0.2511 (2)	−0.0445 (8)	0.3014 (3)	0.0568 (18)	
H13	0.2727	−0.0324	0.2755	0.068*	
C14	0.2155 (2)	0.0556 (8)	0.2940 (3)	0.0551 (16)	
H14	0.2126	0.1346	0.2625	0.066*	
C15	0.18380 (19)	0.0406 (6)	0.3324 (2)	0.0408 (12)	
H15	0.1594	0.1094	0.3273	0.049*	
C16	0.17531 (15)	−0.0050 (5)	0.5079 (2)	0.0270 (9)	
C17	0.21857 (16)	0.0542 (6)	0.5187 (2)	0.0330 (10)	
H17	0.2353	0.0500	0.4856	0.040*	
C18	0.23708 (18)	0.1196 (7)	0.5781 (2)	0.0459 (13)	
H18	0.2662	0.1603	0.5851	0.055*	
C19	0.2125 (2)	0.1245 (6)	0.6267 (2)	0.0461 (13)	
H19	0.2250	0.1692	0.6668	0.055*	
C20	0.17016 (19)	0.0650 (7)	0.6171 (2)	0.0426 (12)	
H20	0.1539	0.0677	0.6507	0.051*	
C21	0.15130 (17)	0.0009 (6)	0.5579 (2)	0.0346 (10)	
H21	0.1221	−0.0390	0.5514	0.042*	
C22	0.13970 (14)	−0.2882 (5)	0.4482 (2)	0.0276 (9)	
C23	0.16943 (15)	−0.3689 (6)	0.4943 (2)	0.0349 (10)	
H23	0.1946	−0.3173	0.5178	0.042*	
C24	0.16261 (19)	−0.5276 (6)	0.5068 (3)	0.0406 (12)	
H24	0.1832	−0.5823	0.5377	0.049*	
C25	0.12537 (18)	−0.6007 (6)	0.4730 (3)	0.0430 (12)	
H25	0.1206	−0.7065	0.4809	0.052*	
C26	0.0947 (2)	−0.5208 (7)	0.4275 (3)	0.0487 (14)	
H26	0.0692	−0.5721	0.4050	0.058*	
C27	0.10177 (16)	−0.3649 (6)	0.4152 (2)	0.0360 (10)	
H27	0.0809	−0.3107	0.3845	0.043*	
Cl2	0.5000	0.0930 (15)	0.2500	0.288 (5)*	
Cl3	0.44248 (15)	0.3008 (6)	0.2579 (2)	0.0923 (12)*	0.50
C28	0.5000	0.2911 (17)	0.2500	0.101 (4)*	
H28A	0.5054	0.3360	0.2094	0.122*	0.50
H28B	0.5205	0.3360	0.2870	0.122*	0.50

### Atomic displacement parameters (Å^2^)

**Table:**

	*U*^11^	*U*^22^	*U*^33^	*U*^12^	*U*^13^	*U*^23^
Au	0.02219 (10)	0.02619 (11)	0.03374 (10)	0.00001 (6)	0.00111 (7)	0.00359 (7)
Cl1	0.0923 (13)	0.0844 (13)	0.0850 (12)	0.0519 (11)	0.0347 (10)	0.0537 (11)
S1	0.0244 (5)	0.0319 (6)	0.0480 (6)	0.0006 (5)	0.0041 (5)	0.0140 (5)
P1	0.0215 (5)	0.0231 (5)	0.0295 (5)	−0.0008 (4)	0.0022 (4)	0.0022 (4)
O1	0.0297 (17)	0.0335 (18)	0.0484 (19)	0.0048 (14)	0.0108 (14)	0.0135 (15)
N1	0.0287 (19)	0.037 (2)	0.040 (2)	0.0057 (17)	0.0116 (16)	0.0095 (18)
C1	0.029 (2)	0.031 (2)	0.030 (2)	0.0004 (18)	0.0043 (17)	0.0032 (18)
C2	0.022 (2)	0.037 (2)	0.037 (2)	0.0010 (19)	0.0071 (17)	0.011 (2)
C3	0.044 (3)	0.043 (3)	0.037 (2)	0.009 (2)	0.007 (2)	0.006 (2)
C4	0.047 (3)	0.055 (3)	0.034 (2)	0.003 (3)	0.004 (2)	0.010 (2)
C5	0.040 (3)	0.048 (3)	0.059 (3)	0.016 (2)	0.013 (3)	0.027 (3)
C6	0.060 (4)	0.043 (3)	0.056 (3)	0.020 (3)	0.024 (3)	0.008 (3)
C7	0.050 (3)	0.047 (3)	0.038 (2)	0.014 (3)	0.018 (2)	0.011 (2)
C8	0.035 (3)	0.041 (3)	0.058 (3)	0.003 (2)	0.016 (2)	0.021 (3)
C9	0.053 (3)	0.044 (3)	0.082 (4)	0.009 (3)	0.026 (3)	0.030 (3)
C10	0.029 (2)	0.032 (2)	0.0255 (19)	−0.0056 (19)	0.0037 (17)	−0.0043 (18)
C11	0.035 (2)	0.044 (3)	0.036 (2)	0.002 (2)	0.0049 (19)	−0.004 (2)
C12	0.032 (3)	0.075 (4)	0.043 (3)	−0.001 (3)	0.010 (2)	−0.017 (3)
C13	0.047 (3)	0.088 (5)	0.039 (3)	−0.023 (3)	0.021 (3)	−0.021 (3)
C14	0.069 (4)	0.062 (4)	0.037 (3)	−0.021 (3)	0.017 (3)	0.003 (3)
C15	0.045 (3)	0.040 (3)	0.037 (2)	−0.007 (2)	0.009 (2)	0.004 (2)
C16	0.026 (2)	0.023 (2)	0.031 (2)	0.0017 (17)	0.0029 (17)	0.0024 (17)
C17	0.026 (2)	0.035 (3)	0.037 (2)	−0.0047 (19)	0.0043 (18)	0.000 (2)
C18	0.040 (3)	0.047 (3)	0.046 (3)	−0.013 (3)	−0.005 (2)	−0.003 (3)
C19	0.064 (3)	0.039 (3)	0.032 (2)	−0.005 (3)	0.000 (2)	0.000 (2)
C20	0.052 (3)	0.044 (3)	0.033 (2)	−0.002 (2)	0.010 (2)	−0.002 (2)
C21	0.031 (2)	0.038 (3)	0.036 (2)	−0.001 (2)	0.0072 (19)	0.001 (2)
C22	0.028 (2)	0.022 (2)	0.034 (2)	−0.0022 (17)	0.0084 (17)	0.0012 (17)
C23	0.028 (2)	0.032 (3)	0.043 (2)	−0.0005 (19)	0.0030 (19)	0.004 (2)
C24	0.042 (3)	0.031 (3)	0.051 (3)	0.011 (2)	0.016 (2)	0.010 (2)
C25	0.056 (3)	0.023 (2)	0.055 (3)	−0.002 (2)	0.023 (3)	0.002 (2)
C26	0.054 (4)	0.035 (3)	0.058 (3)	−0.015 (3)	0.013 (3)	−0.009 (3)
C27	0.037 (2)	0.032 (3)	0.037 (2)	−0.006 (2)	0.001 (2)	0.000 (2)

### Geometric parameters (Å, °)

**Table:**

Au—P1	2.2578 (11)	C12—H12	0.9400
Au—S1	2.3064 (11)	C13—C14	1.371 (10)
Cl1—C5	1.740 (5)	C13—H13	0.9400
S1—C1	1.753 (5)	C14—C15	1.381 (8)
P1—C22	1.801 (5)	C14—H14	0.9400
P1—C16	1.814 (5)	C15—H15	0.9400
P1—C10	1.816 (4)	C16—C17	1.392 (6)
O1—C1	1.358 (5)	C16—C21	1.394 (6)
O1—C8	1.447 (6)	C17—C18	1.387 (7)
N1—C1	1.259 (6)	C17—H17	0.9400
N1—C2	1.402 (6)	C18—C19	1.380 (8)
C2—C7	1.382 (7)	C18—H18	0.9400
C2—C3	1.393 (6)	C19—C20	1.368 (8)
C3—C4	1.386 (7)	C19—H19	0.9400
C3—H3	0.9400	C20—C21	1.382 (7)
C4—C5	1.372 (8)	C20—H20	0.9400
C4—H4	0.9400	C21—H21	0.9400
C5—C6	1.372 (9)	C22—C23	1.381 (6)
C6—C7	1.390 (7)	C22—C27	1.396 (6)
C6—H6	0.9400	C23—C24	1.411 (7)
C7—H7	0.9400	C23—H23	0.9400
C8—C9	1.516 (7)	C24—C25	1.372 (8)
C8—H8A	0.9800	C24—H24	0.9400
C8—H8B	0.9800	C25—C26	1.386 (9)
C9—H9A	0.9700	C25—H25	0.9400
C9—H9B	0.9700	C26—C27	1.388 (8)
C9—H9C	0.9700	C26—H26	0.9400
C10—C11	1.385 (7)	C27—H27	0.9400
C10—C15	1.389 (7)	Cl2—C28	1.701 (18)
C11—C12	1.383 (7)	Cl3—C28	1.797 (5)
C11—H11	0.9400	C28—H28A	0.9800
C12—C13	1.379 (9)	C28—H28B	0.9800
			
P1—Au—S1	178.01 (4)	C14—C13—C12	120.8 (5)
C1—S1—Au	102.58 (16)	C14—C13—H13	119.6
C22—P1—C16	104.4 (2)	C12—C13—H13	119.6
C22—P1—C10	107.0 (2)	C13—C14—C15	120.2 (6)
C16—P1—C10	105.3 (2)	C13—C14—H14	119.9
C22—P1—Au	112.37 (14)	C15—C14—H14	119.9
C16—P1—Au	112.79 (15)	C14—C15—C10	119.8 (5)
C10—P1—Au	114.24 (15)	C14—C15—H15	120.1
C1—O1—C8	116.4 (4)	C10—C15—H15	120.1
C1—N1—C2	121.4 (4)	C17—C16—C21	118.9 (4)
N1—C1—O1	120.5 (4)	C17—C16—P1	123.7 (4)
N1—C1—S1	126.7 (4)	C21—C16—P1	117.4 (3)
O1—C1—S1	112.8 (3)	C18—C17—C16	120.3 (5)
C7—C2—C3	118.1 (4)	C18—C17—H17	119.9
C7—C2—N1	120.1 (4)	C16—C17—H17	119.9
C3—C2—N1	121.6 (5)	C19—C18—C17	119.7 (5)
C4—C3—C2	120.6 (5)	C19—C18—H18	120.2
C4—C3—H3	119.7	C17—C18—H18	120.2
C2—C3—H3	119.7	C20—C19—C18	120.7 (5)
C5—C4—C3	119.8 (5)	C20—C19—H19	119.6
C5—C4—H4	120.1	C18—C19—H19	119.6
C3—C4—H4	120.1	C19—C20—C21	120.0 (5)
C6—C5—C4	121.2 (5)	C19—C20—H20	120.0
C6—C5—Cl1	119.3 (5)	C21—C20—H20	120.0
C4—C5—Cl1	119.5 (5)	C20—C21—C16	120.4 (5)
C5—C6—C7	118.6 (5)	C20—C21—H21	119.8
C5—C6—H6	120.7	C16—C21—H21	119.8
C7—C6—H6	120.7	C23—C22—C27	118.9 (4)
C2—C7—C6	121.8 (5)	C23—C22—P1	122.2 (3)
C2—C7—H7	119.1	C27—C22—P1	118.9 (3)
C6—C7—H7	119.1	C22—C23—C24	121.0 (4)
O1—C8—C9	106.3 (4)	C22—C23—H23	119.5
O1—C8—H8A	110.5	C24—C23—H23	119.5
C9—C8—H8A	110.5	C25—C24—C23	118.9 (5)
O1—C8—H8B	110.5	C25—C24—H24	120.5
C9—C8—H8B	110.5	C23—C24—H24	120.5
H8A—C8—H8B	108.7	C24—C25—C26	120.9 (5)
C8—C9—H9A	109.5	C24—C25—H25	119.5
C8—C9—H9B	109.5	C26—C25—H25	119.5
H9A—C9—H9B	109.5	C25—C26—C27	119.9 (5)
C8—C9—H9C	109.5	C25—C26—H26	120.1
H9A—C9—H9C	109.5	C27—C26—H26	120.1
H9B—C9—H9C	109.5	C26—C27—C22	120.4 (5)
C11—C10—C15	119.4 (4)	C26—C27—H27	119.8
C11—C10—P1	122.2 (4)	C22—C27—H27	119.8
C15—C10—P1	118.4 (4)	Cl2—C28—Cl3	92.7 (5)
C12—C11—C10	120.6 (5)	Cl2—C28—H28A	113.2
C12—C11—H11	119.7	Cl3—C28—H28A	113.2
C10—C11—H11	119.7	Cl2—C28—H28B	113.2
C11—C12—C13	119.2 (6)	Cl3—C28—H28B	113.2
C11—C12—H12	120.4	H28A—C28—H28B	110.5
C13—C12—H12	120.4		
			
P1—Au—S1—C1	−143.5 (12)	C12—C13—C14—C15	1.0 (9)
S1—Au—P1—C22	171.4 (12)	C13—C14—C15—C10	−0.1 (9)
S1—Au—P1—C16	53.8 (12)	C11—C10—C15—C14	−1.4 (8)
S1—Au—P1—C10	−66.5 (12)	P1—C10—C15—C14	176.8 (4)
C2—N1—C1—O1	−176.1 (4)	C22—P1—C16—C17	114.7 (4)
C2—N1—C1—S1	5.2 (7)	C10—P1—C16—C17	2.2 (5)
C8—O1—C1—N1	2.4 (7)	Au—P1—C16—C17	−123.0 (4)
C8—O1—C1—S1	−178.8 (4)	C22—P1—C16—C21	−67.0 (4)
Au—S1—C1—N1	170.6 (4)	C10—P1—C16—C21	−179.5 (4)
Au—S1—C1—O1	−8.2 (4)	Au—P1—C16—C21	55.3 (4)
C1—N1—C2—C7	−121.0 (5)	C21—C16—C17—C18	−0.8 (7)
C1—N1—C2—C3	64.5 (7)	P1—C16—C17—C18	177.5 (4)
C7—C2—C3—C4	0.7 (8)	C16—C17—C18—C19	0.5 (8)
N1—C2—C3—C4	175.3 (5)	C17—C18—C19—C20	0.3 (9)
C2—C3—C4—C5	−0.3 (8)	C18—C19—C20—C21	−0.9 (9)
C3—C4—C5—C6	−0.1 (9)	C19—C20—C21—C16	0.6 (8)
C3—C4—C5—Cl1	−179.0 (5)	C17—C16—C21—C20	0.2 (7)
C4—C5—C6—C7	0.1 (10)	P1—C16—C21—C20	−178.1 (4)
Cl1—C5—C6—C7	178.9 (5)	C16—P1—C22—C23	−31.3 (4)
C3—C2—C7—C6	−0.7 (8)	C10—P1—C22—C23	80.0 (4)
N1—C2—C7—C6	−175.4 (5)	Au—P1—C22—C23	−153.9 (3)
C5—C6—C7—C2	0.4 (9)	C16—P1—C22—C27	146.6 (4)
C1—O1—C8—C9	171.6 (5)	C10—P1—C22—C27	−102.1 (4)
C22—P1—C10—C11	−35.4 (4)	Au—P1—C22—C27	24.0 (4)
C16—P1—C10—C11	75.3 (4)	C27—C22—C23—C24	1.9 (7)
Au—P1—C10—C11	−160.4 (3)	P1—C22—C23—C24	179.9 (4)
C22—P1—C10—C15	146.5 (4)	C22—C23—C24—C25	−1.1 (7)
C16—P1—C10—C15	−102.9 (4)	C23—C24—C25—C26	−0.2 (8)
Au—P1—C10—C15	21.4 (4)	C24—C25—C26—C27	0.6 (9)
C15—C10—C11—C12	1.9 (7)	C25—C26—C27—C22	0.3 (8)
P1—C10—C11—C12	−176.2 (4)	C23—C22—C27—C26	−1.5 (7)
C10—C11—C12—C13	−1.0 (8)	P1—C22—C27—C26	−179.5 (4)
C11—C12—C13—C14	−0.5 (9)		

### Hydrogen-bond geometry (Å, °)

**Table:**

Cg1 is the centroid of the C22–C27 ring.

**Table:**

*D*—H···*A*	*D*—H	H···*A*	*D*···*A*	*D*—H···*A*
C21—H21···N1^i^	0.94	2.55	3.310 (6)	138
C26—H26···S1^ii^	0.94	2.86	3.738 (6)	156
C7—H7···Cg1^i^	0.94	2.96	3.784 (5)	147

Symmetry codes: (i) −*x*, −*y*, −*z*+1; (ii) *x*, *y*−1, *z*.
